# Breast cancer in immigrant women in Europe and Italy: epidemiology, disparities, and pathways to equity

**DOI:** 10.3389/or.2026.1860530

**Published:** 2026-08-19

**Authors:** Lucia Mangone, Isabella Bisceglia, Francesco Marinelli, Annamaria Pezzarossi, Fortunato Morabito, Luca Ghirotto, Amelia Ceci, Guglielmo Ferrari, Elisa Mazzini

**Affiliations:** 1 Epidemiology Unit, Azienda USL-IRCCS di Reggio Emilia, Reggio Emilia, Italy; 2 Associazione “Vittorio Lodini per La Ricerca in Chirurgia” di Reggio Emilia, Reggio Emilia, Italy; 3 Qualitative Research Unit, Azienda USL-IRCCS di Reggio Emilia, Reggio Emilia, Italy; 4 Health Promotion Staff and Gender Medicine Group, Azienda USL-IRCCS di Reggio Emilia, Reggio Emilia, Italy; 5 Scientific Directorate, Azienda USL-IRCCS di Reggio Emilia, Reggio Emilia, Italy

**Keywords:** breast cancer, health equity, immigrant women, screening disparities, tumour biology

## Abstract

Immigrant women in Europe and Italy represent a growing and heterogeneous population whose breast cancer (BC) experience is influenced by social, cultural, and healthcare system factors. Although first-generation immigrants generally have a lower BC incidence, risk converges over time with that of native populations, while barriers to care persist. To analyze disparities in BC screening, diagnosis, tumour characteristics, treatment pathways, and outcomes among immigrant women in Europe and Italy, and to identify strategies to promote equity. A narrative review of European and Italian literature was conducted, focusing on screening uptake, stage at diagnosis, tumour biology, access to diagnostics and treatments, and outcomes. Social determinants of health and healthcare system barriers were also considered. Screening participation is consistently lower among immigrant women because of a combination of language barriers, limited knowledge of screening programmes, limited culturally shaped beliefs, about health and cancer, competing work and caregiving responsibilities, and practical obstacles such as transportation and appointment scheduling, leading to delayed diagnosis and more advanced disease. Socioeconomic disadvantage and residence in underserved areas further limit access to diagnostic procedures, treatment, and follow-up. Potential differences in tumour biology have been reported, but data are limited and rarely stratified by region of origin. Disparities also affect treatment pathways, with delays and unequal access to advanced technologies. Long-term outcomes remain poorly documented. Reducing disparities requires coordinated policy and health system interventions, including standardized migration data collection, equitable access to screening and treatment, inclusion in clinical research, and culturally tailored education and patient-navigation programs. Equity should be central to BC care in Europe and Italy.

## Introduction

Breast cancer (BC) remains the most frequently diagnosed malignancy among women worldwide and constitutes a major public-health challenge owing to its high incidence, morbidity, and mortality (1). Recent global estimates indicate that in 2022, there were approximately 2.3 million new cases of female BC and around 670.000 deaths (2). Projections indicate that by 2050, the annual number of new cases could increase by approximately 38%, while mortality could rise by nearly 68% (2). This alarming trend is expected to continue unless there are substantial improvements in early detection, timely access to care, and equitable treatment delivery (2).

Within Europe, BC remains the most commonly diagnosed cancer in women (3,4). In several countries incidence rates exceed 100 per 100,000 women, and age-standardized mortality rates, despite advances in early detection and treatment, remain substantial (3,4). According to the European Cancer Organisation (ECO), more than 12 million women in Europe are currently living with a cancer diagnosis, and BC ranks among the three most common cancers in women, underscoring the magnitude of the public-health burden (5). These data are consistent with estimates from the GLOBOCAN 2022 project, which reported approximately 2.3 million new cases of female BC worldwide in 2022 (4). Immigrant women are a particularly vulnerable population with respect to BC for multiple reasons. First, participation in organized screening programs is consistently lower in immigrant groups, reflecting a combination of language barriers, limited knowledge of screening invitations and procedures, culturally shaped beliefs about health and cancer, competing work and caregiving responsibilities, and practical obstacles such as transportation and appointment scheduling, often exacerbated by residence in underserved areas (6). Second, reduced screening uptake is associated with delayed diagnosis, often resulting in more advanced disease at presentation and adversely affecting treatment options and outcomes. A meta-analysis comparing immigrant and native-born women reported that foreign-born women had a lower likelihood of being diagnosed at a localized stage (OR 0.88; 95% CI 0.82–0.95), indicating a systematic disadvantage in early detection (7). Third, socioeconomic disadvantage, cultural isolation, and residence in rural or marginalized urban settings may further limit timely access to high-quality diagnostic procedures and effective therapeutic interventions (8). Fourth, immigrant and minority women are frequently underrepresented in cancer clinical trials, largely due to limited trial availability, restrictive eligibility criteria, and barriers in the offer and enrolment process. This underrepresentation contributes to persistent gaps in knowledge regarding tumour biology, treatment response, and survivorship in these populations (9,10). Fifth, the intersection of migration status, ethnicity, socioeconomic status, and health system factors may generate compounded disparities that are not fully captured by standard national cancer statistics (11). Finally, emerging—but still limited—evidence suggests that tumour biology features, including hormone-receptor status, HER2 expression, and Ki-67 proliferation index, may differ among immigrant populations, with potential implications for prognosis and treatment decision-making (12). Taken together, these considerations highlight that BC in immigrant women is not only an issue of social equity but also one of clear clinical relevance. Understanding how immigrant status influences screening participation, diagnostic stage, tumour characteristics, access to care, treatment pathways, and outcomes is essential to inform targeted interventions and reduce avoidable disparities. Accordingly, this narrative review aims to synthesize current evidence on BC among immigrant women across Europe and Italy, with particular focus on tumour biology, diagnosis, access to care, treatment pathways, and clinical outcomes. By identifying gaps in existing knowledge and highlighting opportunities for research and policy action, this review seeks to support improved prevention, diagnosis, and management of BC in this vulnerable population. Beyond individual risk factors, the experience of BC among immigrant women is shaped by intersecting social determinants—including gender, socioeconomic position, legal status, racism and discrimination, and the organisation of healthcare systems—that operate across the cancer care continuum. Situating immigrant women within this broader equity framework is essential to understand how structural barriers, rather than inherent biological differences, drive many of the observed disparities.

## Methods

This article is based on a narrative review of the literature on breast cancer among immigrant women in Europe, with a specific focus on Italy. The narrative review approach was chosen to integrate epidemiological evidence, clinical features, and social and health-system determinants, and to allow inclusion of heterogeneous study designs (quantitative, qualitative, and mixed-methods studies), policy reports, and relevant grey literature.

We searched major biomedical databases (e.g., PubMed/MEDLINE, Embase, Web of Science, and Scopus), as well as Google Scholar and selected institutional websites, for articles published in English or Italian up to December 2025. Search terms combined key concepts related to breast cancer (e.g., “breast cancer”, “mammography”, “screening”, “tumour characteristics”) with migration-related terms (e.g., “immigrant*“, “migrant*“, “foreign-born”, “ethnic minority”) and geographic descriptors (“Europe”, names of European countries, and “Italy”).

We prioritised peer-reviewed original studies and reviews reporting data on: (i) breast cancer incidence, (ii) participation in screening programmes, (iii) stage at diagnosis and tumour characteristics, (iv) access to diagnostics and treatment pathways, and (v) outcomes among immigrant or foreign-born women compared with native-born populations. Studies were included if they provided explicit definitions of migration or foreign-born status, or if relevant information could be inferred from the text. We also considered selected policy documents and reports from European and Italian institutions where they contributed essential contextual information.

Titles and abstracts were screened to identify potentially relevant publications, which were then assessed in full text. Given the narrative nature of the review, no formal risk-of-bias assessment or meta-analysis was conducted. Instead, we summarised and compared findings across studies, highlighting consistencies and discrepancies, and interpreted them in light of broader social determinants of health, healthcare system characteristics, and the specific Italian context.

## Epidemiology and access to screening

Immigrant women—defined as individuals born outside the host country who have relocated for employment, family reunification, asylum, or other migration pathways—constitute a growing and heterogeneous population across Europe and Italy. This heterogeneity extends beyond country or region of origin, to include length of residence, legal status, language proficiency, educational level, employment conditions, and urban versus rural living environments. These intersecting dimensions shape health behaviours, engagement with preventive services, and access to cancer care, and therefore influence participation in screening programmes, stage at diagnosis, treatment pathways, and cancer outcomes.

## Breast cancer incidence in immigrant women: European evidence

European evidence consistently indicates that immigrant women often show a lower incidence of BC compared with native-born populations, although risk convergence is observed in second-generation cohorts or with longer residence. For example, in Sweden, first-generation immigrant women had an incidence rate ratio (IRR) of 0.88 (95% CI 0.86–0.90) versus natives, whereas daughters of immigrants had an IRR of 0.97 (95% CI 0.94–1.01) (13,14). Similarly, a pooled Nordic study reported that non-Western immigrant women had a relative risk of 0.71 (95% CI 0.65–0.78) for BC compared with native women, with risk increasing over time since migration, suggesting acculturation to host-country lifestyle or reproductive risk factors (15).

## Breast cancer incidence among immigrant women in Italy

In Italy, the increasing diversity of the female population due to migration introduces new challenges for cancer prevention and control. Immigrant women are heterogeneous with respect to country of origin, length of stay, language proficiency, socioeconomic integration, and access to health services, and may therefore experience distinct patterns of risk, screening participation, stage at diagnosis, tumour biology, treatment pathways, and outcomes compared with the native Italian population. Data from the Veneto Tumour Registry (2015–19) indicated that female immigrants born in High Migratory Pressure Countries (HMPC) had significantly lower BC incidence compared with Italian-born women (IRR <1 for all HMPC groups), whereas immigrants from Highly Developed Countries did not show this lower incidence (16). Another Italian study reported heterogeneous, origin-specific incidence patterns, with an overall lower incidence among immigrant women that converges toward that of Italian-born women with increasing duration of residence (17).

Beyond incidence patterns, population-based data from Italian screening programmes also point to inequalities in access to organised mammography. A large study conducted in Turin found that immigrant women were less likely to participate in screening even after adjustment for individual-level socioeconomic factors, indicating that migration background exerts an independent effect beyond social disadvantage (18). Regional variation is important in Italy, where screening programmes are organised and implemented at the regional level, with differences in invitation strategies, coverage, and the availability of outreach or mediation services. Immigrant women living in regions with lower screening coverage or fewer culturally tailored initiatives may therefore face compounded disadvantages compared with both Italian-born women in the same region and immigrant women in regions with stronger public health infrastructure (19).

## Access to and uptake of breast cancer screening: European data

Participation in BC screening represents a critical upstream determinant of BC outcomes and is consistently lower among immigrant women across Europe. In Germany, Brzoska et al. reported significantly lower lifetime participation in cancer screening among women with a two-sided migration background compared with non-migrant women, while those with a one-sided migration background showed participation rates closer to those of natives (20). Knowledge gaps further exacerbate these disparities: a German study protocol by Berens et al. found that first-time invitees with migrant backgrounds had substantially poorer understanding of the benefits and harms of mammography, highlighting the combined role of access barriers and health literacy (21). Similarly, Kuehnle et al. demonstrated that migration background and lower educational attainment independently predicted reduced participation in early detection programs, reinforcing the importance of intersecting vulnerabilities (22). Recent scoping reviews further confirm these patterns. Dumky et al. reported wide variation in screening attendance among immigrant women according to country of origin, duration of residence, and sociodemographic factors, while identifying facilitators including culturally tailored outreach, language-appropriate information, and community engagement (23). In Austria, population-based surveys have documented lower mammography uptake among migrant women compared with non-migrants (24). Italian data on screening participation among immigrant women are discussed in more detail in the section above. Likewise, a review by Sterlingova et al. in Nordic countries showed that, despite universal health systems, immigrant women consistently experience lower screening uptake due to language barriers, cultural beliefs, limited health literacy, and structural obstacles such as complex appointment procedures (25).

## Neighbourhood, ethnic enclaves, and structural determinants

Neighbourhood-level factors may further influence BC detection and outcomes. Shen et al systematically reviewed 34 U.S. studies assessing the impact of ethnic enclaves on cancer outcomes, including BC (26). Findings were mixed: some studies associated residence in high-ethnic-density neighbourhoods with a later stage at diagnosis and lower screening participation, particularly among Hispanic and Asian women, reflecting limited healthcare access and language barriers. Conversely, other studies suggested improved survival in enclaves characterised by strong social cohesion and community support, potentially mitigating stress and promoting treatment adherence. Overall, enclave effects appeared highly context-dependent, varying according to socioeconomic conditions, healthcare accessibility, and degree of acculturation. Although derived largely from U.S. data, these observations are relevant to European and Italian settings, where migrant concentration, language isolation, and socioeconomic deprivation may similarly influence BC outcomes and call for spatially informed public-health strategies. Qualitative evidence highlights the barriers to screening among immigrant women. In focus groups conducted by Gold et al. among Somali- and Ethiopian-born women overdue for mammography in Seattle, participants reported language barriers, limited understanding of screening, competing work and family responsibilities, and practical issues such as transport and appointment scheduling, alongside fear, absence of symptoms, and concern about pain (27). Religious beliefs influenced decision-making among Somali participants, while Ethiopian women emphasized trust in physicians, highlighting heterogeneity within immigrant groups. Identified facilitators included physician recommendation, community outreach, peer narratives, and culturally tailored oral or visual materials (27). Although such findings have limited generalisability, they mirror European and Italian evidence of lower screening uptake and later-stage diagnoses among immigrant women (23) and point to actionable strategies to enhance early detection.

In summary, immigrant women represent a heterogeneous population whose needs require culturally tailored education, targeted community outreach, and enhanced data collection to ensure equitable access to early detection and optimal BC outcomes. Overall, first-generation immigrants tend to have a lower incidence of BC but substantially reduced participation in screening programs, with risk converging toward that of native populations over time. The consistently lower screening uptake observed across multiple countries reflects a complex interplay of cultural, linguistic, and structural barriers, already described above, underscoring the importance of tailored interventions to improve early diagnosis and long-term outcomes (Table 1).

**TABLE 1:** Cancer incidence and breast cancer screening participation among immigrant populations in Europe and selected high-income countries.

Study (reference)	Country/setting	Cancer type(s)	Population/sample size	Migration definition	Study period	Main outcomes	Key findings (immigrants vs. natives)
Hemminki et al. (13)	Sweden	Multiple cancers (incl. BC)	First- and second-generation immigrant women; ∼600,000 descendants of at least one immigrant parent (0–66 years)	First-generation immigrants (foreign-born) and offspring of immigrants vs. native swedes	NR (registry data up to early 2000s)	Standardised incidence ratios (SIRs) for multiple cancers	Overall cancer incidence slightly lower among offspring of immigrants compared with natives; lower BC incidence in first-generation immigrants; incidence in second-generation women closer to that of natives
Hemminki and Li (14)	Sweden	Multiple cancers (incl. BC)	Second-generation immigrant women vs. Swedish-born women	Daughters of immigrants vs. native-born	NR	SIRs for multiple cancers	Cancer risks in second-generation immigrants approach those of natives; BC incidence closer to native levels than in first-generation women
Lamminmäki et al. (15)	Denmark, Finland, Iceland, Norway (nordic countries)	Breast, lung, colorectal	All women registered as residents in national population registries	Non-western immigrants defined as women born outside nordic countries, western/Southern europe, north America, Australia, New Zealand	1986–2019 (Denmark), 1973–2017 (Finland), 1986–2020 (Iceland), 1990–2015 (Norway)	Age-standardised incidence and mortality; rate ratios (RR)	Non-western immigrant women have lower BC incidence than natives (RR 0.71, 95% CI 0.65–0.78); risk increases with duration of residence; mortality patterns vary by cancer type
Ferroni et al. (16)	Veneto, Italy	All cancers and selected sites (incl. BC)	All incident cancer cases in the veneto tumour registry, women classified by area of birth	Country of birth grouped into: Italy, highly developed countries (HDC), high migratory pressure countries (HMPC: Eastern europe, asia, africa, south-central America)	2015–2019	Age-standardised incidence rates; incidence rate ratios (IRR)	Female immigrants from HMPC show significantly lower BC incidence (IRR <1) compared with Italian-born women; women from HDC show incidence similar to natives
Collatuzzo et al. (17)	Italy (sicily)	All cancers and BC	Migrant and non-migrant cancer cases recorded in the regional registry	Migrants defined by country of birth outside Italy; analyses stratified by region of origin	2004–2019	Proportionate morbidity ratios (PMR); odds ratios (OR) for specific cancers	Overall lower cancer incidence in migrants; origin-specific differences; BC incidence in migrant women generally lower, with convergence toward italians over time since migration
Brzoska and abdul-rida (19)	Germany	Cancer screening (multiple sites, incl. BC)	11,709 women in a population-based survey	Women with two-sided migration background (non-German nationality, immigrated after birth, or two foreign-born parents); one-sided migration background; non-migrants	NR (survey year NR)	Lifetime participation in cancer screening	Women with two-sided migration background have significantly lower participation in cancer screening than non-migrants; one-sided migrants closer to natives
Berens et al. (20)	Germany (westphalia-lippe)	BC screening	∼17,000 women aged 50 invited to mammography screening	Women with and without migrant background invited to screening	NR (study protocol)	Informed choice and knowledge about mammography (protocol)	Protocol for assessing informed choice in first-time invitees; highlights concerns about lower knowledge and potential lower uptake in migrant women
Kuehnle et al. (21)	Germany	BC screening/early detection	2,145 women with primary BC treated in six breast cancer centres (out of 3,047 eligible)	Migration background defined by place of birth/nationality (details NR in summary)	2012–2016	Participation in early detection programmes; education; migration background	Both migration background and lower education are independently associated with reduced participation in early BC detection programmes
Dumky et al. (22)	Europe (multiple countries)	BC screening	Immigrant women across european screening programmes (scoping review; sample NR)	Migrant/immigrant women as defined in primary studies	NR	Attendance, knowledge, barriers, facilitators	Wide variation in BC screening attendance by country of origin, years since migration, and sociodemographic factors; key facilitators include culturally tailored
​	​	​	​	​	​	​	outreach, language-appropriate information, community engagement
Dumky et al. (22)	Europe (multiple countries)	BC screening	Immigrant women across european screening programmes (scoping review; sample NR)	Migrant/immigrant women as defined in primary studies	NR	Attendance, knowledge, barriers, facilitators	Wide variation in BC screening attendance by country of origin, years since migration, and sociodemographic factors; key facilitators include culturally tailored outreach, language-appropriate information, community engagement
Wahidie et al. (23)	Austria	BC screening (mammography)	5,118 women aged ≥45 years from the austrian health interview survey	Migrant vs. non-migrant women based on country of birth/nationality (as per survey)	NR (recent survey round)	Participation in mammography screening	Migrant women show significantly lower mammography uptake than non-migrants, even within a universal healthcare system
Di girolamo et al. (18,24)	Turin, Italy	BC screening	Women aged 50–69 invited to organised screening in turin; sample size NR in text (city-wide cohort)	Immigrant vs. Italian-born women, with stratification by socioeconomic position (SEP)	2010–2019	Participation, recall, cancer detection rates; role of SEP	Immigrant women have significantly lower participation in organised screening than Italian-born women, even after adjustment for individual-level SEP; contextual (area-level) inequalities also evident
Sterlingova et al. (25)	Nordic countries	BC screening	Women in mammography screening programmes in nordic countries (systematic review)	Immigrant/migrant women vs. non-migrants, as defined in primary studies	NR	Participation in mammography; factors influencing attendance	Immigrant women consistently show lower screening uptake despite universal health systems; barriers include language, cultural beliefs, limited health literacy, and organisational obstacles
Shen et al. (26)	United States	Multiple cancers (incl. BC)	Immigrant women and other racial/ethnic minorities living in ethnic enclaves (34 studies)	Residence in high-ethnic-density neighbourhoods/enclaves	2000–2023	Stage at diagnosis, screening participation, treatment, survival	Mixed effects of ethnic enclaves: In some groups, higher ethnic density associated with later stage and lower screening; in others, better survival due to social cohesion; mechanisms relevant for understanding neighbourhood effects in europe
Gold et al. (27)	United States (seattle)	BC screening	Somali- and ethiopian-born immigrant women overdue for mammography (focus groups)	Foreign-born women from Somalia or Ethiopia, living in the US, overdue for BC screening	NR	Barriers and facilitators to mammography	Reported barriers include language, limited understanding of screening, competing responsibilities, logistical issues (transport, appointments), fear, no symptoms, concern about pain; facilitators include physician recommendation, community outreach, and culturally tailored materials
Mackenbach et al. (Cited as europe, 2023)*	Europe (multiple countries)	Not focused on incidence; screening and risk factors	Immigrant women in european systems (details NR)	Immigrant/migrant status as defined by primary data sources	NR	Determinants of screening and risk factors	Highlights the role of religious beliefs, cultural norms, and language barriers as key determinants of screening and preventive behaviours among immigrant women in europe

Abbreviations: BC, breast cancer; HMPC, high migratory pressure countries; HDC, highly developed countries; SIR, standardised incidence ratio; IRR, incidence rate ratio; RR, rate ratio; OR, odds ratio; SEP, socioeconomic position; NR, not reported.

## Tumour characteristics, treatment pathways, access to care, and outcome

Differences in screening participation and access to healthcare contribute not only to delayed diagnosis but also to variations in tumour characteristics and, ultimately, clinical outcomes among immigrant women. Several studies indicate that immigrant women are more frequently diagnosed at later stages compared with native populations. A recent meta-analysis reported that migrant women were significantly less likely to be diagnosed with early-stage BC with an odds ratio (OR) of 0.78 (95% CI 0.70–0.87) compared with non-migrants (28). These delays are multifactorial and reflect lower participation in screening programmes, difficulties navigating healthcare services, and limited awareness of BC symptoms. Tumour biological characteristics among immigrant women remain under-investigated; however, emerging evidence suggests potential differences across migration backgrounds. In Norway, a population-based cohort study demonstrated a lower incidence of luminal A-like BC among immigrant women from Sub-Saharan Africa (IRR 0.43, 95% CI 0.28–0.66), South-East Asia (IRR 0.63, 95% CI 0.51–0.79), South Asia (IRR 0.67, 95% CI 0.52–0.86), and Eastern Europe (IRR 0.86, 95% CI 0.76–0.99) compared with non-immigrants (12). Similarly, a population-based study from the Netherlands reported a higher likelihood of high-grade tumours among migrant women than among native women; notably, this difference was attenuated in women who participated in organised screening programmes (29). Despite these observations, the literature rarely provides detailed stratification by oestrogen and progesterone receptor status (ER/PR), HER2 expression, and Ki-67 proliferation index according to migration background. Socioeconomic factors, duration of residence, region of origin, area of residence (urban versus rural), and language proficiency may all influence tumour stage and biology, though supporting evidence remains limited (30,31).

In addition, legal status and associated entitlements to healthcare, experiences of discrimination, and varying degrees of social support and community cohesion can modulate both the timeliness of presentation and the ability to complete complex treatment pathways. Grouping all immigrant women together therefore risks obscuring important differences between, for example, recent asylum seekers, long-term labour migrants, and second-generation women born in the host country.

Across Europe, migration background and social deprivation consistently emerge as key determinants of diagnostic and biological disparities (23). While screening and early detection have been central to disparity research, less attention has been paid to inequities along the treatment pathways. Immigrant women may experience delays in surgery, systemic therapy initiation, radiotherapy, and follow-up care due to linguistic and cultural barriers, socioeconomic constraints, and challenges navigating healthcare systems (32). Access to comprehensive diagnostic evaluation, including immunohistochemical assessment of ER, PR, HER2, and Ki-67, is essential for optimal management, yet disparities in access to these services persist across Europe (33,34). Migrant populations face additional barriers, as limited health literacy, language difficulties, and unfamiliarity with the healthcare system increase the likelihood that eligible patients may not receive timely biomarker testing, thereby potentially compromising stage-appropriate treatment decisions and outcomes (33). Evidence from outside Europe suggests that such disparities are not inevitable. Fatiregun et al. investigated whether immigration status influenced receipt of guideline-concordant treatment among women with stage 1–2 HER2-positive or triple-negative BC in Ontario, Canada (35). After adjustment for demographic and clinical factors, no significant differences were observed between immigrants and long-term residents, suggesting that within a well-functioning universal healthcare system, immigration status did not limit access to optimal therapy. These findings contrast with much of the European and Italian literature, which continues to document delayed diagnosis and suboptimal treatment among immigrant women. Data on long-term outcomes—including recurrence, survival, quality of life, and treatment adherence—remain limited. Available studies suggest that immigrant women may experience worse outcomes, although results are heterogeneous and often confounded by socioeconomic and healthcare system factors (12,36). In contrast, evidence from settings with equitable access to high-quality diagnostics, timely treatment, and standardized follow-up indicates that such disparities can be substantially reduced. Collectively, these findings suggest that the inequities observed in Europe and Italy are largely driven by modifiable systemic and social determinants rather than intrinsic biological risks associated with immigrant status.

In the Italian context, multilevel analyses from the Umbria region have shown that area-level deprivation and age influence the stage at diagnosis for breast and other common cancers (30). Although migration background was not the primary focus of these studies, the concentration of immigrant populations in more deprived urban and peri-urban areas suggests that immigrant women may be disproportionately exposed to contextual factors associated with later-stage presentation. Systematically integrating migration-related variables into Italian cancer registry data would allow these hypotheses to be tested and help disentangle the effects of individual- and area-level deprivation among immigrant and Italian-born women.

Table 2 summarises key BC disparities affecting immigrant women, encompassing screening, tumour features, access to diagnostics, treatment pathways, and outcomes, and highlights the role of socioeconomic and structural barriers in shaping these inequities.

**TABLE 2:** Tumour characteristics, access to care, treatment pathways, and outcomes among immigrant women with breast cancer.

Study (reference)	Country/setting	Population/sample and period	Migration definition	Study design	Tumour characteristics/outcomes	Key findings (immigrants vs. natives)
Harvey-sullivan et al. (28)	Europe/multiple countries	Meta-analysis of migrants vs. non-migrants with cancer (incl. BC), 2000–2023	Migrants vs. non-migrants, as defined in primary studies	Meta-analysis	Stage at diagnosis	Migrant women have lower odds of early-stage BC diagnosis (OR 0.78, 95% CI 0.70–0.87), indicating more advanced stage at presentation compared with non-migrants
Hjerkind et al. (12)	Norway	Women aged 20–75 and 20–49 years, 2005–2015	Immigrant women grouped by broad WHO region of birth (sub-saharan africa, south-east asia, south asia, eastern europe) vs. non-immigrants	Population-based cohort	BC subtype-specific incidence (e.g., luminal A-like)	Immigrant women from sub-saharan africa, south-east asia, south asia, and eastern europe show lower incidence of luminal A-like BC (IRR 0.43–0.86) compared with non-immigrants; subtype distribution differs by region of origin
Dassen et al. (29)	Netherlands (rotterdam)	Women diagnosed with BC in rotterdam, 2012–2015	Migrant vs. native women (definition based on country of birth/parents’ origin)	Observational, registry-based study	Tumour grade; impact of screening attendance	Migrant women more often diagnosed at younger age and with higher-grade tumours; among women who participated in organised screening, differences in tumour characteristics were attenuated, suggesting a levelling effect of screening
Vercelli et al. (30)	Umbria, Italy	Women with BC (and other cancers) in the umbria cancer registry, 2001–2010	Migration status not primary focus; analysis by area-level deprivation	Multilevel observational study	Stage at diagnosis by deprivation level	Higher area-level deprivation associated with later stage at diagnosis for BC (and other cancers); suggests that women in deprived areas, where immigrants are often concentrated, may face contextual barriers to early diagnosis
Koçak and çiçek gümüş (31)	Turkey	589 women (Turkish n = 302, syrian immigrants n = 287)	Syrian immigrants vs. Turkish citizens	Cross-sectional comparative study	Knowledge about early diagnosis; perceived BC risk	Syrian immigrant women have lower knowledge about early diagnosis and different perceptions of BC risk compared with Turkish women, indicating important information gaps that may delay detection
Dumky et al. (22)	Europe (multiple countries)	Immigrant women in european BC screening programmes (scoping review)	Immigrant/migrant women as defined in primary studies	Scoping review	Stage at diagnosis; access to screening and diagnostic services	Across european studies, immigrant women are more often diagnosed at later stages and face multiple barriers to timely diagnosis and treatment, including language, cultural, and structural obstacles
Aleshire et al. (32)	United States of America (south-east region)	39 black women (including immigrants)	Racial/ethnic minority women; immigration status variably reported	Retrospective qualitative study	Access to mammography; treatment delays; perceived outcomes	Women report access barriers (costs, transport, system complexity) and delays in surgery/systemic therapy; these factors contribute to worse outcomes, especially in socioeconomically disadvantaged and minority groups
Normanno et al. (33)	Multiple european countries	Patients with solid tumours (incl. BC); numbers and period NR	Migrant status not always explicit; focus on health-system variation	Multicountry survey/observational study	Access to and quality of biomarker testing (ER, PR, HER2, Ki-67, NGS)	Marked inequalities in access to high-quality biomarker testing across europe; language barriers, health-literacy issues, and system organisation can disproportionately affect migrants’ access to precision oncology
European cancer patient coalition (34)	Europe	Cancer patients in europe; report based on multiple data sources	Migrant status not systematically captured	Policy report/advocacy document	Access to precision medicine and biomarker testing	Highlights structural barriers to equitable biomarker testing and precision oncology; underscores the need for better data on migration status when evaluating access to advanced diagnostics
Fatiregun et al. (35)	Ontario, Canada	Women aged 18–75 with stage 1–2 HER2-positive or triple-negative BC, diagnosed 2012–2019 and followed to 2023	Legal immigrants vs. long-term residents, based on immigration registry	Retrospective cohort	Guideline-concordant treatment; overall survival	In a universal healthcare system, legal immigrant women receive guideline-concordant treatment at rates comparable to long-term residents and do not have increased risk of death, suggesting that equity is achievable when access barriers are minimised
Bhargava et al. (36)	Norway	Immigrant and non-immigrant women invited to BreastScreen Norway	Immigrants defined by country of birth (e.g., Pakistan, Somalia) vs. Norwegian-born	Registry-based study/trial	BC-specific survival among screening invitees	Some immigrant groups show worse BC-specific survival despite participation in screening, pointing to the role of post-diagnosis factors (treatment, follow-up, social and structural determinants)

Abbreviations: BC, breast cancer; ER, oestrogen receptor; PR, progesterone receptor; HER2, human epidermal growth factor receptor 2; Ki-67, proliferation index; NGS, next-generation sequencing; IRR, incidence rate ratio; OR, odds ratio; SES, socioeconomic status; NR, not reported.

## Implications for Italy and Europe

The evidence summarized above has important implications for policy, clinical practice, and research across Europe, and specifically in Italy. These findings underscore that migration should be considered alongside other axes of social stratification—such as income, education, and area-level deprivation—when designing and evaluating cancer control strategies. Addressing disparities in breast cancer among immigrant women therefore requires multilevel interventions that act at the levels of communities, healthcare organisations, and national policy, rather than focusing solely on individual behaviours.

First, the consistently lower participation in BC screening and the delayed diagnosis observed among immigrant women—which reflect the combination of linguistic, cultural, informational, and structural barriers described above—underscore the need for targeted outreach strategies, culturally tailored education, and structured navigational support in multiple languages.

Second, healthcare systems must ensure equitable access not only to screening programs but also to timely, high-quality diagnostic services and personalized treatment, in order to prevent downstream disparities in outcomes. Third, cancer registries and surveillance systems should systematically collect migration-related variables—including country of origin, length of residence, language proficiency, socioeconomic status, and area of residence (urban versus rural)—to enable more accurate risk stratification, monitoring outcomes, and evaluation of targeted interventions. Wherever feasible, monitoring systems should also disaggregate data by key migration-related dimensions, including (at a minimum) broad region of origin (e.g., EU, non-EU, specific macro-areas), duration of residence in the host country (e.g., recent versus long-term), and legal status (where this can be collected ethically and within legal constraints), and should incorporate indicators of language proficiency and socioeconomic position (such as education, employment status, and area-level deprivation). Collecting and analysing data along these axes would allow identification of sub-groups of immigrant women at highest risk of late diagnosis or under-treatment and support the design of targeted, context-specific policies and interventions, rather than assuming that all immigrant women share the same needs and barriers. Fourth, further research is needed to characterise tumour biology in immigrant populations, including molecular subtypes and biomarkers. This would help determine whether clinically meaningful differences exist and how these may interact with access to care.

For Italy, where a universal healthcare system is organised at the regional level, these recommendations translate into several concrete priorities. Regional cancer plans and breast screening programmes could explicitly include migration and equity objectives, such as monitoring invitation and participation rates by country of birth and length of residence, expanding the use of cultural mediators and interpreters in screening and diagnostic services, and developing partnerships with community organisations that represent major migrant groups. Particular attention should be paid to regions and local health authorities with lower screening coverage and higher levels of social deprivation, where immigrant women are more likely to face combined social and organisational barriers along the breast cancer care pathway.

**FIGURE 1:**
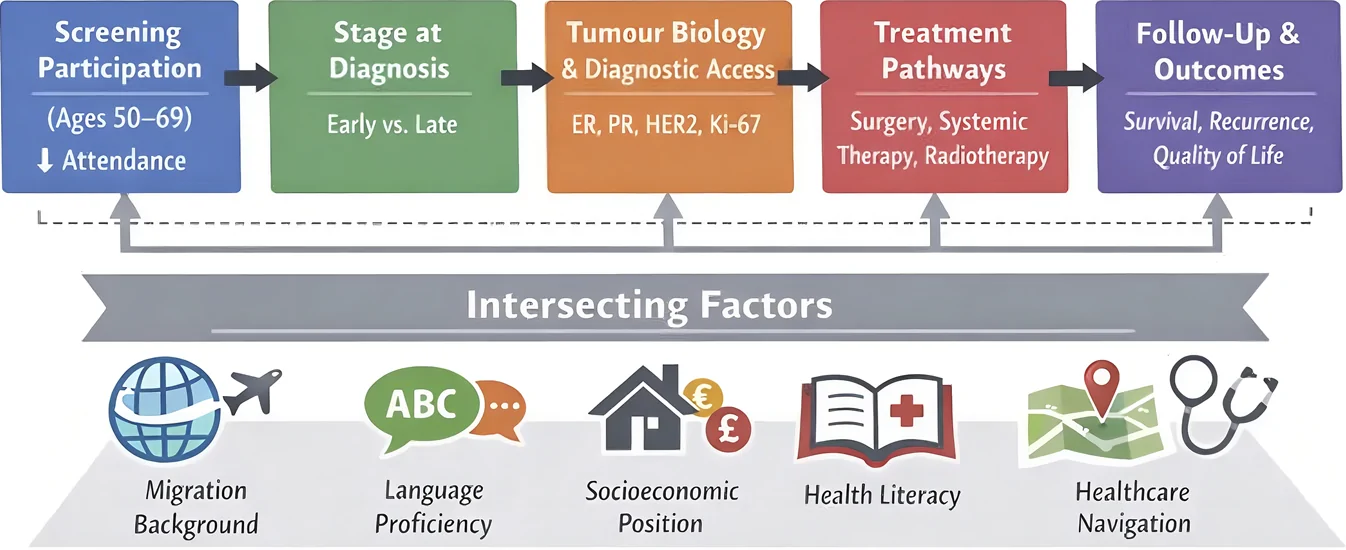
Conceptual framework of breast cancer care for immigrant women. The figure illustrates the sequential determinants of breast cancer outcomes across the continuum of care, from screening participation to follow-up and survivorship. Participation in organised screening programs—restricted to the target age group (50–69-year)—represents the initial and most influential step, directly shaping the stage at diagnosis. Stage at diagnosis interacts with tumour biology and access to timely diagnostic testing (including ER, PR, HER2, and Ki-67 assessment) to determine treatment pathways. Adherence to therapy and engagement in follow-up care ultimately influence survival, recurrence, and quality of life. Structural, cultural, and individual factors—such as migration background, language proficiency, socioeconomic position, health literacy, and healthcare navigation—act across all stages, generating cumulative advantages or disadvantages for immigrant women.

Finally, effective reduction of disparities will require multilevel interventions that integrate community engagement, primary care involvement, culturally competent communication, health system navigation, and supportive policy initiatives. Such approaches should be embedded within national cancer control plans and adapted to local healthcare contexts. Figure 1 conceptual framework of breast cancer care pathways in immigrant women, spanning screening, diagnosis, treatment, and follow-up. It depicts how individual, cultural, and health system–level factors interact across the continuum of care to influence stage at diagnosis, tumour characteristics, treatment adherence, and long-term outcomes, while also identifying key points for targeted intervention.

## Conclusion and recommendations

Breast cancer among immigrant women represents a growing public health concern in Europe and Italy, reflecting the interplay between migration, social determinants of health, and access to healthcare services. A global overview by Elhawary et al. highlights substantial sociodemographic disparities in female BC incidence and mortality, identifying migration as a key factor shaping both risk and outcomes (37). First-generation immigrants often exhibit a lower initial incidence of BC, however, risk tends to converges with that of host population over time as a result of lifestyle, reproductive, and acculturation-related factors. Concurrently, migration-related barriers—including limited access to screening, and difficulties communicating with and navigating healthcare services already described above—may contribute to delayed diagnosis and increased mortality. These findings are particularly relevant in Europe and Italy, where immigrant women frequently present with more advanced disease. Importantly, observed disparities are not intrinsic to migration status but emerge from modifiable social and structural determinants, emphasizing the need for culturally adapted education, accessible screening programmes, patient navigation, and migration-sensitive registry data. Framing immigrant status as a marker of differential exposure to social, legal, and health-system constraints, rather than as a biological risk factor, helps to shift attention towards modifiable contexts—such as entitlement to care, availability of interpretation services, culturally competent communication, and territorial organisation of screening programmes—that can be changed through policy and organisational reform.

Across Europe and Italy, immigrant women consistently experience lower participation in BC screening, delayed diagnosis, and potential inequities in tumour biology and treatment access. Data on tumour markers—including oestrogen and progesterone receptor status (ER/PR), HER2 expression, and Ki-67 proliferation index—stratified by migration background, region of origin, or duration of residence, remain limited. Similarly, disparities in treatment delivery, including surgery, systemic therapy, radiotherapy, and adherence to clinical guidelines, are insufficiently characterized, while long-term outcomes such as recurrence, survival, and quality of life remain poorly documented. The complex interactions between migration status, area of residence, socioeconomic conditions, and healthcare system factors are also underexplored.

Addressing these gaps requires coordinated policy and practice interventions (19). Cancer registries should systematically collect data on immigration status, country of origin, duration of residence, language proficiency, socioeconomic indicators, and tumour biology to enable robust surveillance and evaluation of disparities. Screening programs should implement culturally tailored outreach, provide interpreter or cultural mediator support, develop multilingual education, and routinely monitor participation by migration status. Diagnostic and treatment pathways must ensure timely and equitable access to high-quality care, supported by patient navigation services and culturally competent multidisciplinary teams. Moreover, active inclusion in clinical trials is essential to ensure that treatment efficacy is assessed across diverse populations.

In conclusion, immigrant women constitute a critical and underserved subpopulation within BC control strategies. Integrating research, health-system reform, and culturally sensitive interventions is essential to reduce avoidable disparities, optimize outcomes, and ensure that equity remains a central objective of BC prevention, diagnosis, and treatment in Europe and Italy.

Future research should prioritize longitudinal cohort studies, enriched and harmonised registry data, biomarker-driven analyses, and interventional studies aimed at improving equity across the entire continuum of BC care.
